# Transcriptome analysis of a social caterpillar, *Drepana arcuata*: De novo assembly, functional annotation and developmental analysis

**DOI:** 10.1371/journal.pone.0234903

**Published:** 2020-06-22

**Authors:** Chanchal Yadav, Myron L. Smith, Jayne E. Yack

**Affiliations:** Department of Biology, Carleton University, Ottawa, Ontario, Canada; Nazarbayev University, KAZAKHSTAN

## Abstract

The masked birch caterpillar, *Drepana arcuata*, provides an excellent opportunity to study mechanisms mediating developmental changes in social behaviour. Larvae transition from being social to solitary during the 3^rd^ instar, concomitant with shifts in their use of acoustic communication. In this study we characterize the transcriptome of *D*. *arcuata* to initiate sociogenomic research of this lepidopteran insect. We assembled and annotated the combined larval transcriptome of “social” early and “solitary” late instars using next generation Illumina sequencing, and used this transcriptome to conduct differential gene expression analysis of the two behavioural phenotypes. A total of 211,012,294 reads generated by RNA sequencing were assembled into 231,348 transcripts and 116,079 unigenes for the functional annotation of the transcriptome. Expression analysis revealed 3300 transcripts that were differentially expressed between early and late instars, with a large proportion associated with development and metabolic processes. We independently validated differential expression patterns of selected transcripts using RT-qPCR. The expression profiles of social and solitary larvae revealed differentially expressed transcripts coding for gene products that have been previously reported to influence social behaviour in other insects (e.g. cGMP- and cAMP- dependent kinases, and bioamine receptors). This study provides the first transcriptomic resources for a lepidopteran species belonging to the superfamily Drepanoidea, and gives insight into genetic factors mediating grouping behaviour in insects.

## Introduction

Sociality is key to the success of many insects [1–3]. The ultimate benefits associated with sociality, including enhanced foraging, predator defense, disease resistance and increased survival, have been well studied (e.g. [1–4]). Proximate mechanisms mediating social behaviours such as group formation, division of labour, and foraging have been studied at different levels of analysis, including hormonal, neural, sensory and genetic (e.g. [5–8]). Over the past two decades, the advent of genomic resources and tools has led to immense progress in the field of sociogenomics, a discipline that focuses on the molecular genetic basis of sociality [9]. Within this field, comparative approaches have led to the identification of genes or gene products associated with social behaviours. One approach is to compare different species or higher-level taxa exhibiting similar or different social behaviours. For example, a comparative sociogenomic analysis using honeybee (*Apis mellifera*), fire ant (*Solenopsis invicta*), and paper wasp (*Polistes metricus*) transcriptomes was conducted to understand the genetic underpinnings of caste development [10]. The results suggested that shared molecular pathways and biological functions mediate caste development in these three eusocial insect lineages. Such studies rely on analyses of genes or pathways that are conserved across the taxa and may not reveal species- or lineage-specific novel genes involved in sociality. An alternative approach is to compare the genetic bases of different social behaviours within a species. Analysis of temporal polyethism (i.e. developmental changes in behaviour) in honeybees (*A*. *mellifera*), where workers transition from nurses to foragers, is a good example of such an intraspecific approach. Using microarray analyses of RNAs expressed in worker bee brains, researchers identified differentially expressed genes associated with age polyethism [11,12]. Similarly, in locusts (*Locusta migratoria*, *Schistocerca gregaria*), more than 200 differentially expressed genes were correlated with the transition between solitary and gregarious phases [13,14]. By comparing within species, we can better understand how differential regulation of a particular gene or set of genes can mediate changes in social behaviours.

**Fig 1:**
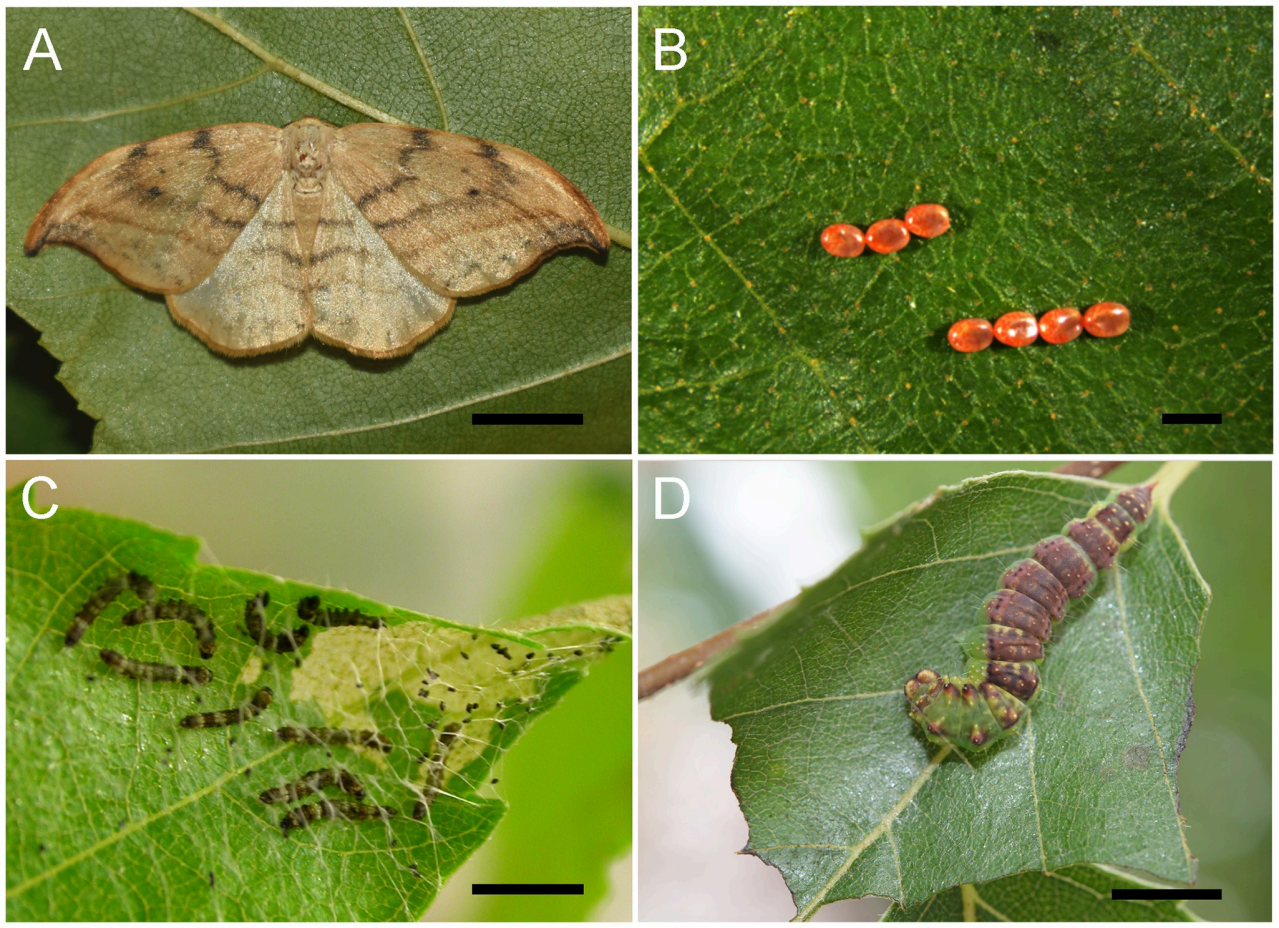
Selected developmental stages of *Drepana arcuata*. (A) Adult moth. Scale bar: 5 mm. (B) Eggs laid in rows. Scale bar: 1 mm. (C) Group of early (I) instars in a silk shelter on a birch (*Betula papyrifera*) leaf. Scale bar: 2 mm. (D) Solitary late (IV) instar in a silk shelter on a birch leaf. Scale bar: 5 mm.

A developmental shift in grouping behaviour is observed in a number of arthropods, including spiny lobsters, spiders, sawfly larvae, and larvae of several lepidopteran species [1,15–18]. Benefits incurred from developmental changes in sociality may be related to shifts in competition for resources, foraging efficiency, thermoregulation, and predator defense [1,19,20]. Proximate mechanisms underlying such ontogenetic shifts are poorly understood, with limited research conducted to date. To the best of our knowledge, no genomic approaches have been undertaken to study developmental shifts in grouping behaviour in arthropods. The masked birch caterpillar (*Drepana arcuata*, Drepanoidea) presents an excellent opportunity to explore such mechanisms. The caterpillar transitions from a social to solitary lifestyle during development ([21]; Fig 1), and the complex acoustic communication systems that mediate these social interactions have been studied in this species and its relatives (e.g. [22–26]). The first two instars (I, II) are referred to as “early instars”, the fourth and fifth (IV,V) as “late instars” and the third (III) as the “transitional instar”. Eggs are laid in rows and neonates form small social groups that exhibit vibratory-mediated social interactions in shared silk shelters [21,25] (Fig 1). Larvae remain in groups until third instar, when they transition to a solitary lifestyle. Late instars exhibit vibratory-mediated territorial behaviour to defend solitary silk leaf shelters [22,24]. We propose the masked birch caterpillar is highly suitable for sociogenomic research because: (i) it transitions in social grouping behaviour at a predictable time—the third instar; (ii) we have insights into the sensory-motor communication mechanisms mediating grouping and solitary behaviour; (iii) there is opportunity to conduct interspecies comparative genomic analyses, as different species of Drepanoidea exhibit varying levels of sociality [27].

This study takes an important step towards developing the masked birch caterpillar as a non-model organism for sociogenomic research, and provides the first genomic resource for any Drepanoidea species. There are three main goals: First, to assemble and annotate the larval transcriptome of *D*. *arcuata*. Second, to use this *de novo* assembled transcriptome as a reference to conduct differential gene expression analysis between “social” early and “solitary” late instars. Third, to identify the expression profiles of genes that are potentially involved in social interactions (i.e. “social” genes).

## Materials and methods

### Sample preparation

**Fig 2:**
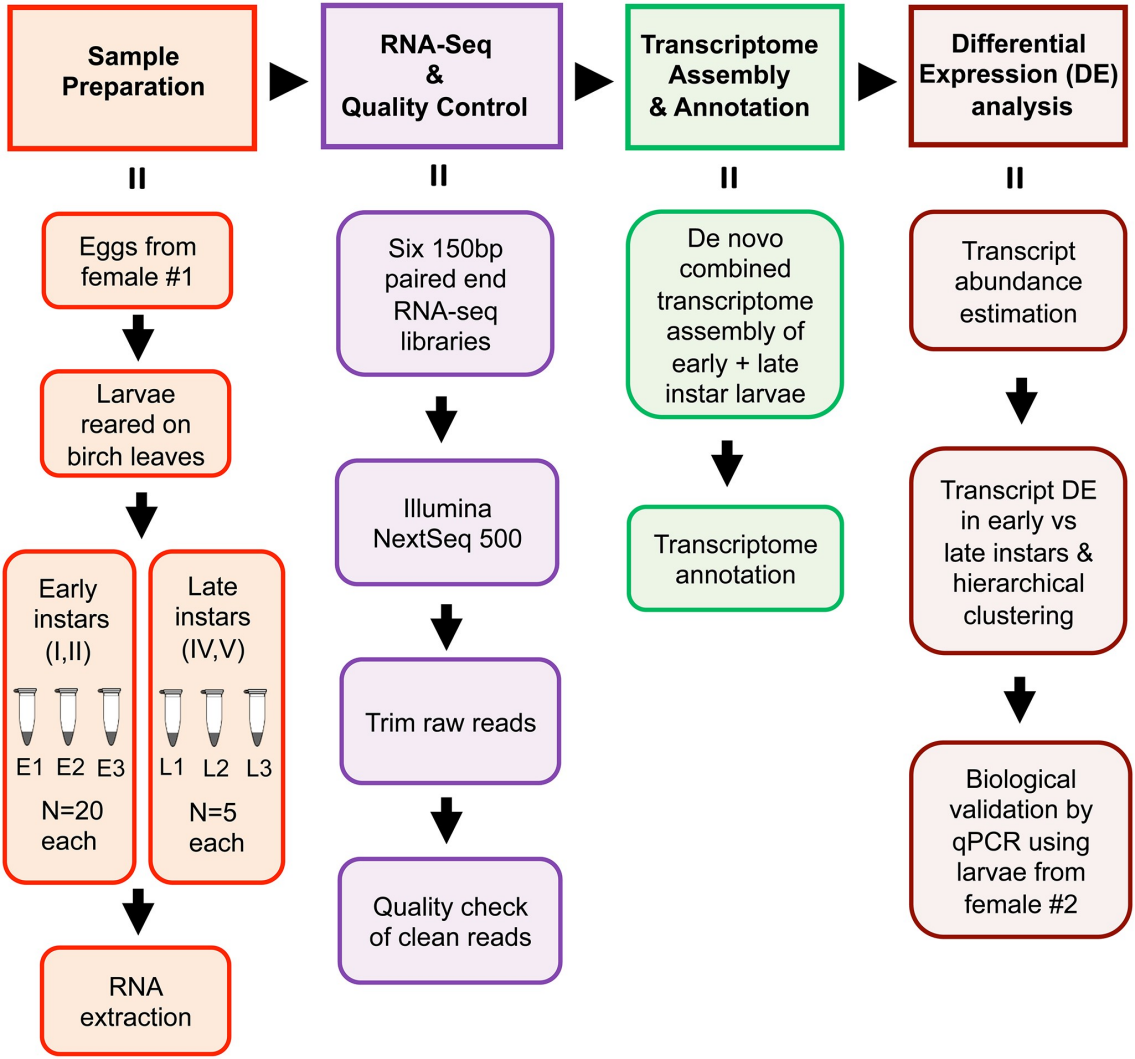
Summary of methods used for transcriptome assembly and differential transcript expression analysis. E1, E2, E3 and L1, L2, L3 refer to the 3 replicates of early and late instars, respectively.

An overview of methods used in this study is shown in Fig 2. Larvae were reared from eggs laid by females captured at ultraviolet lights near Ottawa, ON, Canada (45.4215 °N, 75.6972 °W). Caterpillars were reared on paper birch (*Betula papyrifera*) leaves held in water-filled vials contained in glass jars at room temperature (21–23°C) on a 16 h: 8 h light: dark cycle. Instars were identified based on their head capsule morphology [21]. Early and late instars used for RNA extraction were in groups and solitary in their shelters, respectively, when collected. No specific permits were required for the collection of moths for this study. This study did not involve any protected or endangered species.

For high-throughput transcriptome sequencing, larvae from the same female (female #1, Fig 2) were used for all RNA extractions. Three biological replicates for each of early instars (I, II; N = 20 larvae [a mix of approximately equal numbers of I & II] per replicate) and late instars (IV,V; N = 5 larvae [3:2 mix of IV & V] per replicate) were frozen separately in liquid nitrogen prior to RNA isolation (see below) for sequencing. Early instars are much smaller in body size (1.7–5.5 mm) than late instars (6.6–20 mm) [21] and therefore to obtain sufficient RNA, more early instars were required per replicate.

### RNA extraction and sequencing

Total RNA was extracted using a Norgen Biotek RNA extraction kit (Norgen Biotek Corp, Thorold, ON, Canada) from whole caterpillar bodies that were flash frozen in liquid nitrogen. Extracted RNA samples were assessed for quantity and quality using Qubit HS RNA kit (Thermo Fisher Scientific, Waltham, MA, USA) and Fragment Analyzer High Sensitivity RNA kit (Agilent, Santa Clara, CA, United States), respectively, and subjected to paired-end Illumina sequencing at the StemCore sequencing facility (Ottawa Hospital Research Institute, ON, Canada). At the facility, RNA sequencing libraries were generated using TruSeq RNA Library Prep Kit v2 (Illumina, San Diego, CA, USA) using manufacturer’s protocol. The quality and quantity of each sample library were assessed using Agilent Fragment Analyzer (Agilent, Santa Clara, CA, USA) with the High Sensitivity NGS Fragment Analysis assay and Qubit Double Stranded DNA HS kit (Thermo Fisher Scientific, Waltham, MA, USA), respectively. RNA sequencing was performed on the Illumina NextSeq500 platform to generate 150 bp paired-end reads. Analysis described in the sections below were performed remotely on the Extreme Science and Engineering Discovery Environment (XSEDE) [28].

### De novo transcriptome assembly and annotation

Raw sequences were quality checked using FastQC v0.11.7 (https://www.bioinformatics.babraham.ac.uk/projects/fastqc/) and trimmed for adapter sequences, poor quality, and short sequences (<50 bp) using Trimmomatic-0.36 [29]. The resulting clean reads from both early and late instars were then assembled into transcripts using *de novo* assembler Trinity v2.4.0 [30] with default parameters, including the normalization step.

Using Trinotate v3.1.0 [31], transcripts were annotated with the following databases: non-redundant database (NR), SwissProt, Kyoto Encyclopedia of Genes and Genomes (KEGG), and Clusters of Orthologous Groups for eukaryotes (KOG) using BLASTx at e-value ≤ 1e-5 [32,33]. Transdecoder 3.0.1 (https://github.com/TransDecoder/TransDecoder/wiki) was used to predict the open reading frames (ORFs), retaining the ORFs of ≥100 amino acids in length. Following annotation, the completeness of the transcriptome was evaluated using BUSCO v3.0 (Benchmarking Universal Single-Copy Orthologs) (https://busco.ezlab.org) [34].

### Differential transcript expression analysis

Transcript expression level differences between early (I, II) and late (IV, V) instars was analyzed using differential gene expression tools included within the *de novo* assembler Trinity- v2.4.0, with default parameters. The expression analysis included two steps: (1) transcript abundance estimation using RSEM [35] and; (2) identification and counting of transcripts expressed differentially between early and late instars using edgeR [36], at False Discovery Rate (FDR) ≤ 0.001 and log_2_ fold change ≥ 2, followed by hierarchical clustering based on expression values.

### RT-qPCR validation of differentially expressed transcripts

To validate the transcript expression data generated by RNA-sequencing and analysis, RT-qPCR was performed with 10 arbitrarily selected genes (3 down-regulated and 7 up-regulated in early instar relative to late instar). RNA was extracted from frozen larvae, all derived from a single female (female #2, Fig 2) different than that used in the RNA-seq analyses (N = 10–15 larvae for early instars with approximately equal numbers of instars I, II, N = 1 larva for instar IV or V; three biological replicates for each). First strand cDNA synthesis was performed (cDNA synthesis quick protocol of New England Biolabs Inc., NEB#M0253) using 1 μg RNA, quantified using Nanodrop Spectrophotometer (Thermo Fisher Scientific, Waltham, MA, USA). 100 ng of cDNA was used for each RT-qPCR reaction. Housekeeping gene elongation factor 1A (*ef1a*) was used as a reference. Primers for the 10 tested genes were designed using Primer3Plus [37] and sequences for the primers are provided in S2 Table. RT-qPCRs were performed using SYBR Fast Universal qPCR kit using three technical replicates for each of the three biological replicates, in a total reaction volume of 20 μl (10 μl SYBR mix, 2 μl each of 10 μM forward and reverse primers, 4 μl MQ water and 2 μl of cDNA). qPCR was carried out using the CFX Connect system (Bio-Rad Laboratories Inc., Hercules, CA, USA) with the following thermal cycling conditions: Initial denaturation at 95°C for 3 min followed by 40 cycles of 95°C for 10s, 60°C for 15s and 72°C for 20s.

Information on the data used in this paper can be found under the “Data Availability Statement”, and includes sequencing and assembly data at NCBI Sequence Read Archive (SRA) and DDBJ/EMBL/GenBank, respectively, and the transcript expression data at NCBI’s Gene Expression Omnibus (GEO) [38].

## Results and discussion

### De novo transcriptome assembly and annotation

#### RNA samples and sequencing

A total of 259,401,581 paired end 150 bp raw reads were obtained from the combined RNA samples (Table 1). Quality assessment indicated that the average GC content was 48.5%, with a Phred quality score above 20 for 100% of the bases. Quality filtering and removal of reads below 50 bp resulted in a total of 211,012,294 high quality reads (81.5% of the raw reads, Phred score ≥ 33). Quality filtered reads were pooled from all replicates of early and late instars to generate a combined reference transcriptome assembly (see below).

**Table 1:** Summary of RNA sequencing data and combined early and late instar larval transcriptome.

**Sequencing data**		
Total raw reads	259,401,581	
Total clean reads	211,012,294	
% GC	48	
**Transcriptome assembly statistics**		
	Transcripts	Unigenes
Total number	231,348	116,079
N50 length	2050	1190
Median length	487	333
Average length	1041.92	671.60
Total assembled bases	241,045,801	77,959,215
% GC	42.38	

#### De novo transcriptome assembly

A total of 231,348 transcripts (N50 = 2050; 116,079 unigenes) were generated (Table 1), where 31.58% of the transcripts were above 1 kb (S1 Fig). Unigene here refers to the longest isoform per cluster of transcripts assembled by Trinity that share sequence content. This large number of transcripts relative to unigenes is normal for the Trinity assembler, as there could be a number of biologically relevant isoforms and paralogs [30]. Therefore, instead of using only the longest isoform per gene (unigene) we used all the transcripts for downstream analysis. BUSCO assessment, which provides quantitative assessment of transcriptome completeness in terms of gene content [34], revealed that the assembly was 97.3% complete, with 2.5% fragmentation and 70.1% duplication. Again, high duplication values are expected, as we used all transcripts generated by Trinity for analysis. BUSCO assessment of unigenes indicated only a ~2% duplication rate. Individual transcriptome assemblies were also generated for early and late instars (S3 Table). These were comparable to other larval Lepidoptera transcriptomes in terms of number of transcripts and N50 values (e.g. [39,40]). For a better representation of the complete larval transcriptome we combined the early and late instar sequences to assemble and annotate a reference transcriptome. This reference transcriptome was used to identify stage-specific RNA expression differences between early (social) and late (solitary) instars.

#### Transcriptome annotation

**Fig 3:**
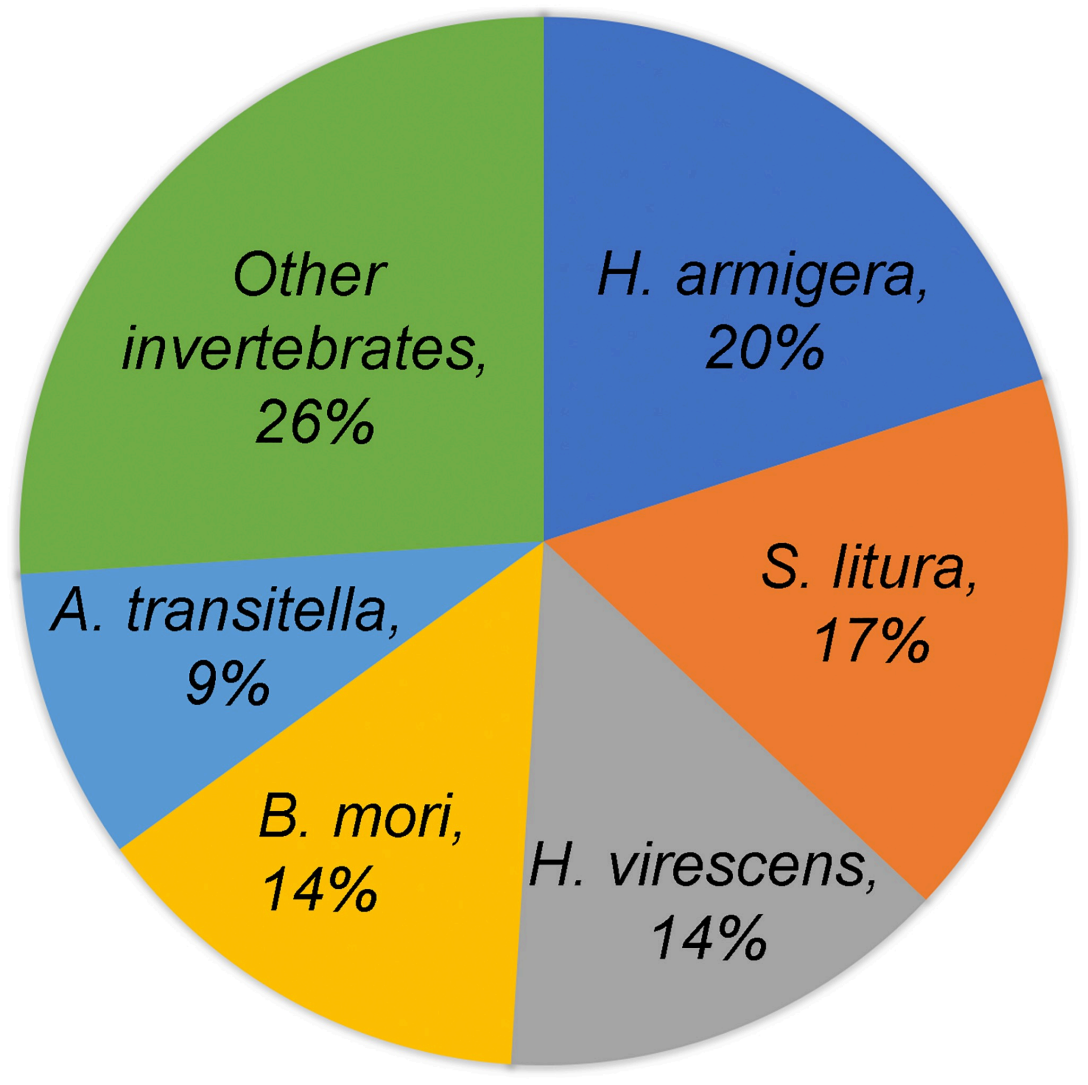
Homology of transcripts to Non-Redundant (NR) protein database. Percentages indicate the distribution of best matches for *D*. *arcuata* transcripts to those of other invertebrates based on the homology search conducted with NR db at an e-value cut off of 1e-5. Top 5 hits were Lepidoptera- *Helicoverpa armigera*, *Spodoptera litura*, *Heliothis virescens*, *Bombyx mori* and *Amyelois transitella*.

**Fig 4:**
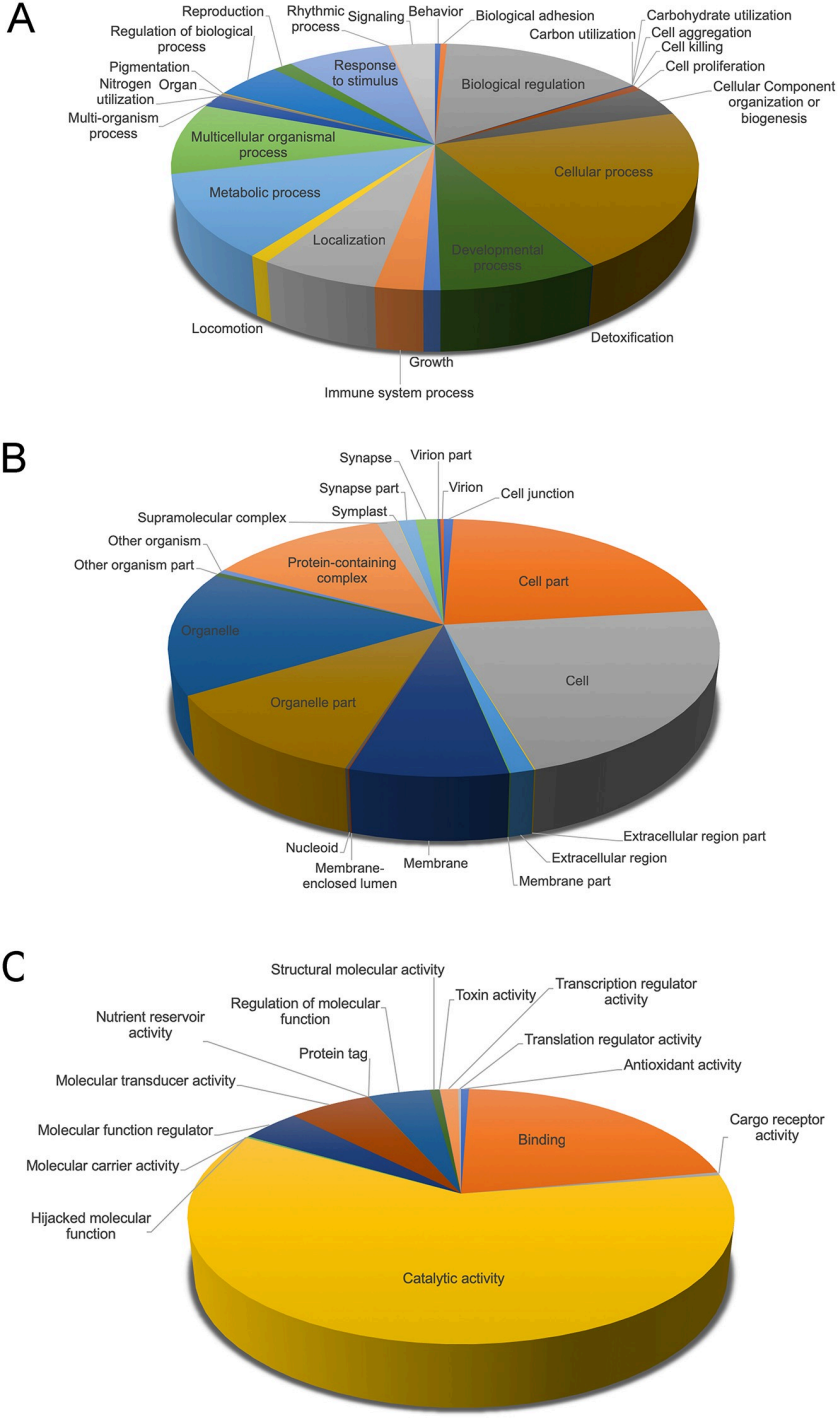
Gene Ontology (GO) classification of larval transcriptome. Transcripts were classified into three functional categories based on GO annotation- (A) biological process, (B) cellular component, and (C) molecular function.

Multiple databases were used to annotate the *de novo* assembled reference transcriptome (see annotation report in S1 File). Using an e-value cut-off of 1e-5, 112,661 transcripts (48.69%) were found to have significant homology to sequences in NCBI’s non-redundant (NR) database. Of these, 74% of transcripts matched best to genes from lepidopteran species and the remaining 26% to other invertebrate species (Fig 3). GO terms, KEGG and KOG annotations were assigned. A total of 75,023 transcripts (32.42%) were assigned to 21,062 GO terms, whereas ~67% of transcripts were of unknown function, highlighting a knowledge gap in our understanding of non-model insect genomes. The major GO categories were biological process (69.87%), cellular component (9.2%), and molecular function (20.85%). Similar to other larval Lepidoptera, biological regulation and metabolic process (in biological process), catalytic activity and binding (in molecular function) and cell and cell part (in cellular component) were amongst the top subcategories represented (Fig 4) (e.g. [39,41]). A total of 19,803 transcripts (8.5%) were annotated to KEGG pathways, divided into 5 categories (S2 Fig), and 49,918 putative proteins (predicted by TransDecoder) were assigned to 4395 KOG terms, classified into 26 KOG groups (S3 Fig). Similar to other larval lepidopterans [39–41] metabolic pathways were most represented in KEGG annotations, and general function only in KOG annotations.

Lepidoptera are one of the largest insect orders, with >157,000 described species [42]. Many species are of economic significance as forest and agricultural pests, and ecologically significant as pollinators and food sources [43]. Additionally, many species are used as model organisms for studies in ecology, evolution and physiological processes (e.g. [44–47]). While genomic information is increasingly being applied to this research, there is a clear lack of genomic resources for most lepidopteran superfamilies [42]. Our study provides the first transcriptomic resources for a species belonging to the superfamily Drepanoidea. The Drepanoidea comprises >1400 species distributed throughout the world [48]. This group includes species of interest for their unique hearing organs [49], as pests of coffee [50], and larval vibroacoustic communication [22–27,51,52]. Notably, due to variability in their social structure both between and within species, and their uniquely complex vibratory communication systems, larval Drepanoidea hold much promise for future research testing hypotheses on the function and evolution of communication and sociality. Our study takes a first step in facilitating this research by providing insights into the larval developmental transcriptome, including the identification of genes potentially associated with a behavioural shift in sociality.

### Differentially Expressed Transcripts (DETs) in early vs late instars

**Fig 5:**
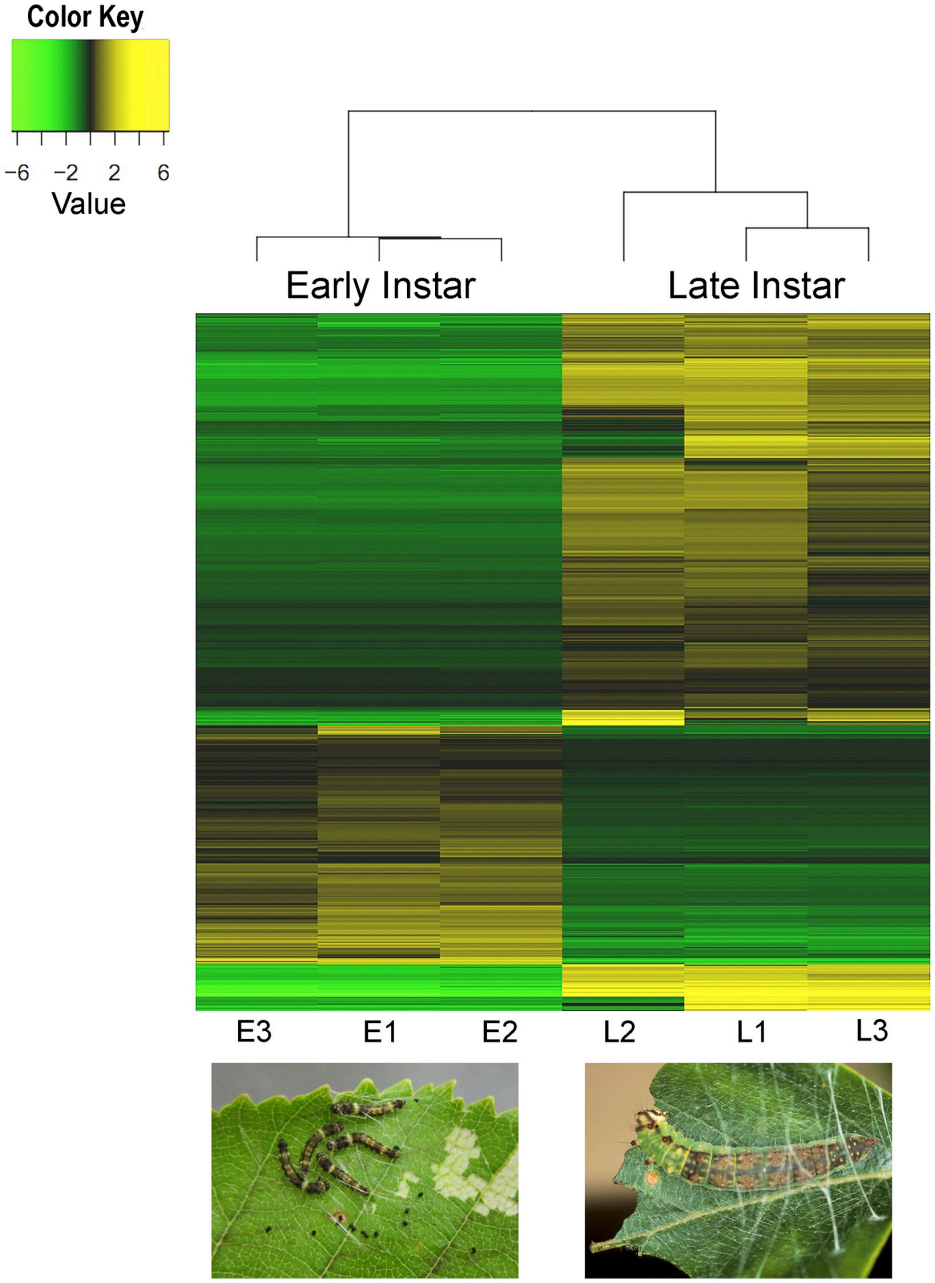
Heat map showing transcripts that are differentially expressed between early and late instars. E1, E2, E3 and L1, L2, L3 refer to the 3 replicates of early and late instars, respectively. Transcripts with differential expression values of log2FC≥2 at FDR≤0.001 were clustered together based on their expression patterns across the samples. The color (see color key on top left corner) indicates low (green) to high (yellow) expression values for transcripts. Dendrograms at top represent the clustering of early and late instar replicates based on their expression.

Based on RNA-seq, 3300 transcripts (3098 unigenes) were identified to be differentially expressed at log_2_FC≥2 (FDR≤0.001) between early and late instars (Fig 5; S2 File). Log_2_FC here refers to the log2 ratio of transcripts’ expression values in late vs early instars. Transcript expression levels based on RNA-seq data were congruent with RT-qPCR results with 10 arbitrarily selected DETs (R = 0.9710; S4 Fig), supporting our use of RNA-seq data to compare gene expression patterns between early and late instars. Of the 3300 DETs, ~34% were down-regulated and ~66% were up-regulated in late instars relative to early instars. Examples of these DETs along with some of the most up-regulated transcripts in early and late instars are listed in Table 2. KEGG pathway analysis revealed that most of the differentially expressed transcripts are involved in metabolic pathways (40.14%), with carbohydrate metabolism, lipid metabolism, and amino acid metabolism representing the most represented subcategories. Also, these pathways were mostly upregulated in late instars relative to early instars (S5 Fig). Metabolic processes were one of the top 3 represented categories in GO annotation as well (S6 Fig). Differential expression of transcripts involved in metabolic processes could relate to developmental differences between early and late instars in mobility, feeding, acoustic signalling, silk production and nest building behaviours [21].

**Table 2:** Some of the most up-regulated transcripts and other interesting DETs in early and late instars.

Instars	*D*. *arcuata* Transcript ID	NR ID	NR ID annotation description	log_2_FC*
Early	TRINITY_DN21660_c3_g1_i1	XP_022817426.1	juvenile hormone esterase-like	15.58
	TRINITY_DN22418_c3_g1_i31	XP_021188341.1	ribosome biogenesis protein WDR12 homolog	13.38
	TRINITY_DN17012_c1_g1_i1	XP_012552749.1	proteoglycan Cow	13.10
	TRINITY_DN22359_c0_g1_i6	XP_021189772.1	cytosolic carboxypeptidase 4-like isoform X1	12.18
	TRINITY_DN20585_c1_g1_i5	XP_022815245.1	histone-lysine N-methyltransferase trithorax isoform X2	12.16
	TRINITY_DN17657_c3_g4_i1	XP_004923731.1	ommochrome-binding protein	10.02
Late	TRINITY_DN20910_c0_g1_i5	XP_013165599.1	PREDICTED**: aminopeptidase N-like isoform X1	-4.25
	TRINITY_DN19503_c2_g2_i2	OWR48843.1	Serine protease H51	-6.42
	TRINITY_DN15158_c4_g1_i10	XP_013188410.1	PREDICTED**: cytochrome c oxidase subunit 6b-2-like	-7.40
	TRINITY_DN20861_c0_g1_i5	XP_021194029.1	mucin-5AC-like isoform X2	-9.74
	TRINITY_DN20396_c0_g1_i4	KOB76727.1	Cytochrome P450 6B46	-10.26
	TRINITY_DN15186_c1_g1_i2	XP_021188290.1	gamma-glutamyltranspeptidase 1-like isoform X2	-13.55
	TRINITY_DN18525_c2_g1_i1	NP_001040518.1	S-phase kinase-associated protein	-13.94
	TRINITY_DN21922_c0_g1_i1	XP_022831709.1	fatty acid synthase-like isoform X2	-14.39
	TRINITY_DN14155_c2_g1_i3	XP_013187092.1	PREDICTED**: glucose dehydrogenase [FAD, quinone]-like	-14.40
	TRINITY_DN21096_c3_g1_i4	NP_001139414.1	urbain precursor	-17.66

* +log_2_FC values indicate upregulation in early instar, and -ve log_2_FC values indicate upregulation in late instar.

**predicted function of respective (NR ID) homolog.

Early instars ‘skeletonize’ birch leaves, feeding on the tender tissue between leaf veins, whereas late instars ingest entire sections of leaf. These feeding differences may account for our observation that digestive enzymes (e.g. *mucin*, *serine protease)* are among the relatively up-regulated DETs in late instars (Table 2). DETs encoding detoxification-related enzymes (*cytochrome P450*, *aminopeptidase N*, *cytochrome C oxidase*) were also up-regulated in late instars relative to early instars (Table 2). Detoxification enzymes are induced in insects in response to plant allelochemicals (e.g. [53,54]). An increased expression of transcripts coding for the detoxification enzymes in late instars could be associated with increased feeding, hence enhanced detoxification of plant secondary metabolites and other toxins. As *D*. *arcuata* larvae develop they change in body color, from brownish-black in early to green in late instars [21]. These striking coloration differences between early and late instars may relate to observed differential expression of pigmentation genes. For example, one of the DETs, *ommochrome-binding protein*, is suggested to influence larval body coloration in the silkworm, *Bombyx mori* [55,56] (Table 2).

#### Sociality-related genes

One of the goals of this study was to assess if there was differential expression of candidate genes for sociality between early and late instars. As defined by Fitzpatrick et al. [57], candidate genes are those that have been identified to influence a certain phenotype in one organism, and are then tested for influencing a similar phenotype in another organism. Several orthologous genes appear to influence social behaviours across taxa, including, but not limited to, foraging, cooperative group living, mating and parental care. Some of the most studied “social” genes are listed in Table 3, of which seven were identified among DETs between early and late instars of *D*. *arcuata*. Of particular interest are cGMP-dependent protein kinases, and biogenic amine-octopamine and dopamine receptors. Although a number of predicted “social” genes were identified among the *D*. *arcuata* DETs, there were some that were not observed to be significantly differentially expressed between early and late instars. These included syntaxin 1a, neuropeptide receptor (Npr)-, corazonin receptor, and vitellogenin receptor genes (Table 3).

**Table 3:** Examples of social genes/molecular products in invertebrates and their expression in larval *D*. *arcuata* transcriptome.

Gene/Product	Organism	*D*. *arcuata* transcript ID	log_2_FC*
octopamine receptor	*L*. *migratoria* [58]	TRINITY_DN15936_c1_g2_i1	4.90
dopamine receptor	*L*. *migratoria* [59]	TRINITY_DN21054_c1_g2_i1	4.86
takeout	*L*. *migratoria* [60]	TRINITY_DN19810_c0_g1_i3	4.82
period	*A*. *mellifera* [61]	TRINITY_DN20311_c0_g1_i6	4.51
neuropeptide F precursor	*Drosophila melanogaster* [62]	TRINITY_DN13765_c0_g1_i2	3.73
npr-1	*Caenorhabditis elegans* [63]	TRINITY_DN16480_c1_g1_i2	0.56**
corazonin receptor	*Harpegnathos saltator* [64]	TRINITY_DN18580_c1_g2_i6	-0.198**
syntaxin 1a	*Lasioglossum albipes* [65]	TRINITY_DN21083_c1_g1_i14	-0.96**
vitellogenin, vitellogenin receptors	*A*. *mellifera* [66]	TRINITY_DN14655_c2_g1_i1	-4.10**
cAMP-dependent protein kinase	*S*. *gregaria* [67]	TRINITY_DN13079_c0_g1_i1	-7.15
*for*, cGMP-dependent protein kinase	*D*. *melanogaster* [68] *A*. *mellifera* [69]	TRINITY_DN18355_c0_g1_i6	-9.61

* +log_2_FC values indicate upregulation in early instar, and -ve log_2_FC values indicate upregulation in late instar.

** indicates log_2_FC values at FDR>0.001, hence not included among DETs.

cGMP-dependent protein kinase encoded by foraging (*for*) gene is implicated in the mediation of social and foraging behaviours in diverse insect orders including Hymenoptera, Orthoptera, and Diptera (Table 3). The natural allelic variation in the *for* gene influences fly phenotypes (rover *vs*. sitter), and the differential expression of the *for* gene is associated with nursing-foraging worker phenotypes in honeybees, ants and wasps, and gregarious-solitary phenotypes in desert locusts. For example, in honeybees there is an increased expression of cGMP-dependent kinases (encoded by *for* gene) in foragers that go out of the hive relative to the nurse bees that stay in the hive. Similarly, fly larvae that express the rover phenotype (carrying rover allele at *for* gene) forage over longer distances than sitters (carrying sitter allele at *for* gene). In *D*. *arcuata*, we observed relative upregulation of transcripts coding for the *for* ortholog in late instars (log_2_FC = -9.969, Table 3). As the *D*. *arcuata* larvae develop from early to late instars, they disperse from the communal silk shelters to forage individually and establish solitary leaf shelters. The refore, the relative increase expression of cGMP-dependent kinase in late instars could be correlated with this change in foraging pattern, similar to what has been observed in rover phenotypes in flies and honeybee foragers [68,69].

Other interesting DETs to highlight are those coding for the biogenic amine receptors, dopamine (DA) and octopamine (OA). Biogenic amines have long been known to influence sociality and related behaviours in different insects such as locusts, ants, and honeybees [58,59,70,71]. Particularly in locusts, some OA and DA receptors have been found to be associated with gregarious-solitary state transitions [58,59,72]. In *D*. *arcuata* larvae, we observed a relative upregulation of both OA and DA receptors in early instars that live in groups, relative to the late instars that live solitarily (Table 3). This differential expression of OA and DA could be associated with group formation and maintenance in early instars, similar to specific DA and OA receptors that mediate gregarious behaviour in locusts.

Our transcriptome analysis thus provides leads into genes that may influence the developmental shift from social to solitary in *D*. *arcuata* larvae, and the putative role of these genes can be further tested using pharmacological and/or genetic techniques such as RNAi and CRISPR. RNAi and CRISPR have been used with insects to study genetic modulation of different behaviours, including gregarious behaviour in the desert locust [67], nurse to forager transition in honeybees [73], migration in the monarch butterfly [74], and sociality in the ponerine ant [75,76].

## Conclusion

This study provides the reference transcriptome for larval *D*. *arcuata*. As the first transcriptomic resource for a species within the superfamily Drepanoidea, this research fills a knowledge gap in the field of Lepidoptera genomics. Also, differential transcript analysis using RNA-seq data from social “early” and solitary “late” instars revealed a marked shift in the transcriptome profile, including changes in the expression of sociality-related genes involved in foraging and grouping behaviours in other insects. The masked birch caterpillars and their relatives, that vary in social grouping behaviour within and between species, offer a great opportunity to explore proximate mechanisms of sociality in larval insects.

## Supporting information

S1 Fig Length distribution of the larval *D*. *arcuata* transcripts assembled by Trinity. The x-axis indicates the length of transcripts (bp) and y-axis indicates the total number of transcripts for each given size range. Out of 231,348 transcripts, ~49% transcripts are above 500 bp, ~17.5% between 500 bp-1kb, and ~31.5% above 1kb in length.
(TIF)

S2 Fig Representation of partitioning of *D*. *arcuata* transcripts into KEGG pathways. OS = Organismal systems, CP = Cellular Processes, EIP = Environmental Information Processing, GIP = Genetic Information Processing, M = Metabolism. The numbers on the right side of bars indicate the total number of transcripts annotated to respective KEGG pathway (labelled on the left), and x-axis shows the percentages for each pathway calculated from the total number of transcripts annotated to KEGG pathways. Metabolic processes were most represented (~26.52%), with carbohydrate, lipid and amino acid metabolism being the top pathways in this group.
(TIF)

S3 Fig Histogram representing classification of clusters of orthologous groups (KOG) for the larval transcriptome. Among the 26 KOG function groups, the transcripts ‘General function prediction only’ was most represented (~16.68%) followed by ‘Signal transduction mechanisms’ (~13.35%). Percentages were calculated for each function class by dividing the total number of transcripts annotated to each class by the total number of transcripts annotated to KOG.
(TIF)

S4 Fig Biological validation of RNA-seq-based DETs using RT-qPCR. Scatter plot showing transcript expression in terms of log2-fold changes obtained from RNA-seq and RT-qPCR data for arbitrarily selected DETs. Each diamond represents a DET, N = 10. Linear regression analysis revealed high correlation between RNA-seq and RT-qPCR data (R^2^ = 0.9429, R = 0.9710).
(TIF)

S5 Fig Representation of partitioning of DETs into KEGG pathways. OS = Organismal systems, CP = Cellular Processes, EIP = Environmental Information Processing, GIP = Genetic Information Processing, M = Metabolism. Maximum number of DETs were identified to be associated with metabolic pathways, within which carbohydrate metabolism (~8.5%) and amino acid metabolism (~5.8%) were the top two pathways.
(TIF)

S6 Fig Gene Ontology (GO) classification of differentially expressed transcripts between early and late instar *D*. *arcuata*. Pie chart represents the classification of transcripts in the functional category—biological process.
(TIF)

S1 Table Availability of data used in the transcriptomic analysis of larval *D*. *arcuata*. (DOCX)

S2 Table Transcript IDs, NR IDs and sequences of primers used for RT-qPCR validation of selected DETs in late vs. early instars. (DOCX)

S3 Table Summary of RNA sequencing data and transcriptome assembly for early and late instar larvae. (DOCX)

S1 File Trinotate annotation report for larval *D*. *arcuata* transcriptome. (ZIP)

S2 File Differential expression information on DETs (log_2_FC≥2; FDR≤0.001) identified between early and late instars. (TXT)
