# Supplementary figures and images for: Transcriptome analysis of a social caterpillar, Drepana arcuata: De novo assembly, functional annotation and developmental analysis
Source: PLoS One. 2020 Jun 22;15(6):e0234903. doi: 10.1371/journal.pone.0234903 (PMC7307738; doi:10.1371/journal.pone.0234903)

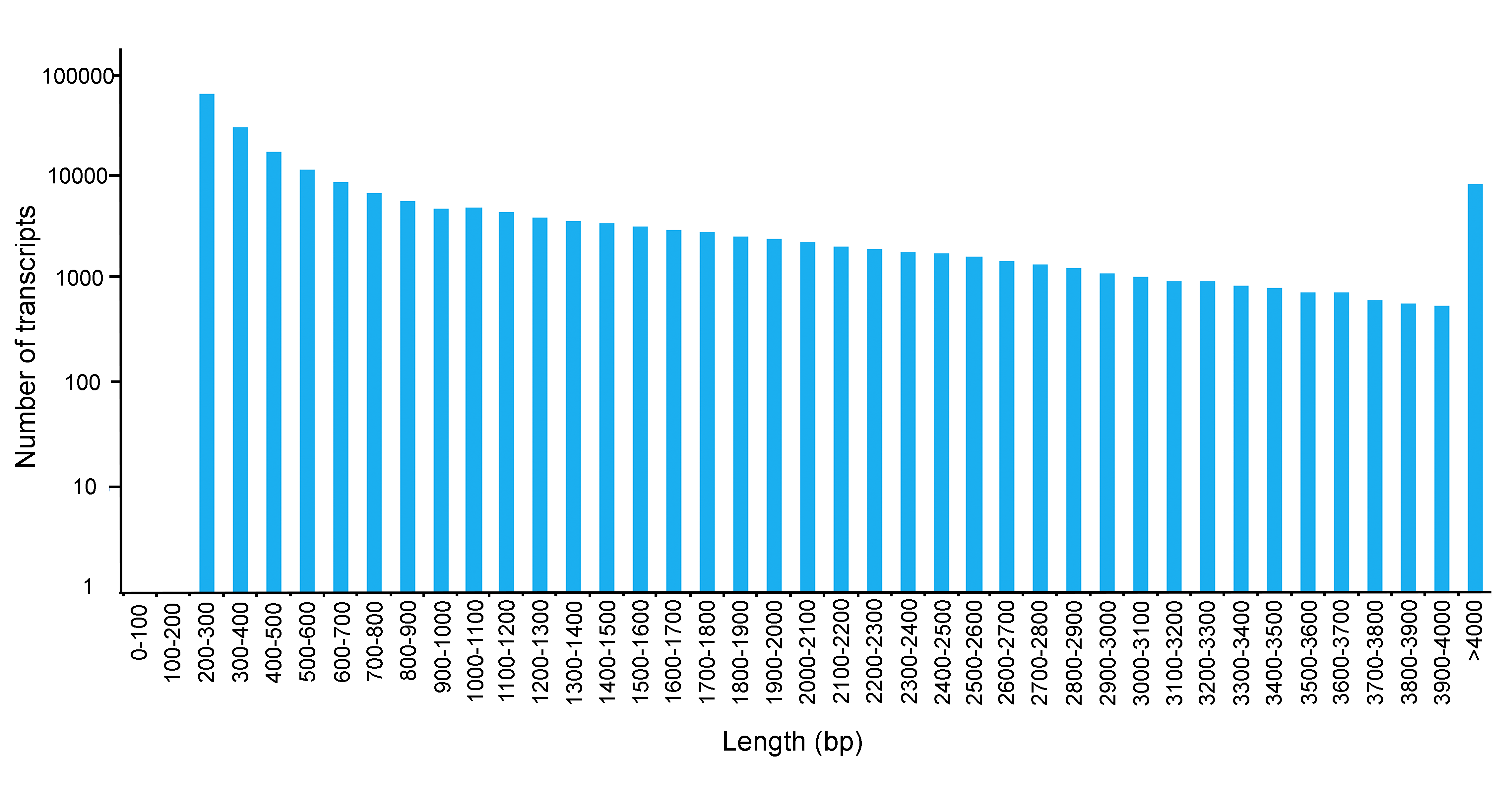

Supplement: S1 Fig — The x-axis indicates the length of transcripts (bp) and y-axis indicates the total number of transcripts for each given size range. Out of 231,348 transcripts, ~49% transcripts are above 500 bp, ~17.5% between 500 bp-1kb, and ~31.5% above 1kb in length. (TIF) [file pone.0234903.s001.tif]

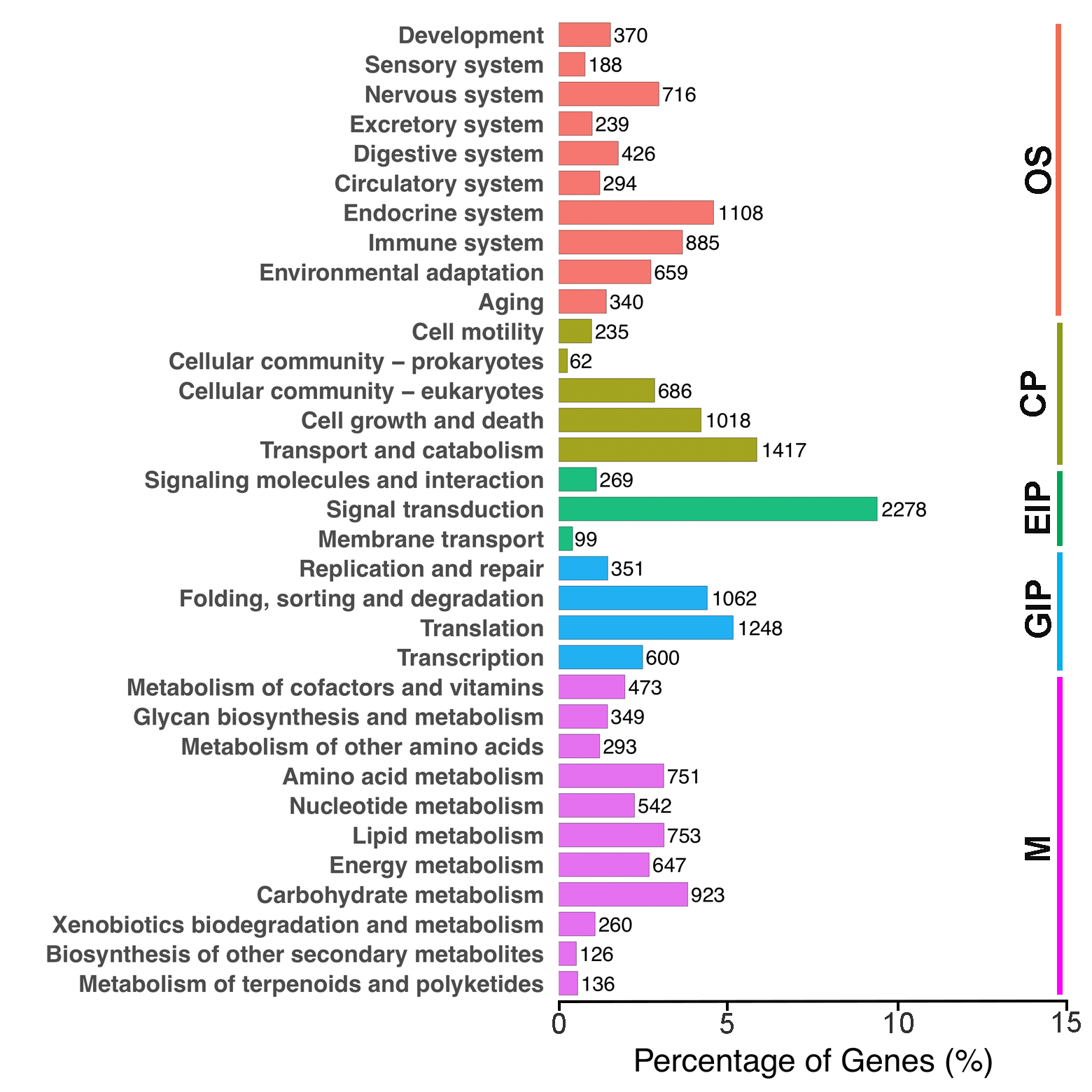

Supplement: S2 Fig — OS = Organismal systems, CP = Cellular Processes, EIP = Environmental Information Processing, GIP = Genetic Information Processing, M = Metabolism. The numbers on the right side of bars indicate the total number of transcripts annotated to respective KEGG pathway (labelled on the left), and x-axis shows the percentages for each pathway calculated from the total number of transcripts annotated to KEGG pathways. Metabolic processes were most represented (~26.52%), with carbohydrate, lipid and amino acid metabolism being the top pathways in this group. (TIF) [file pone.0234903.s002.tif]

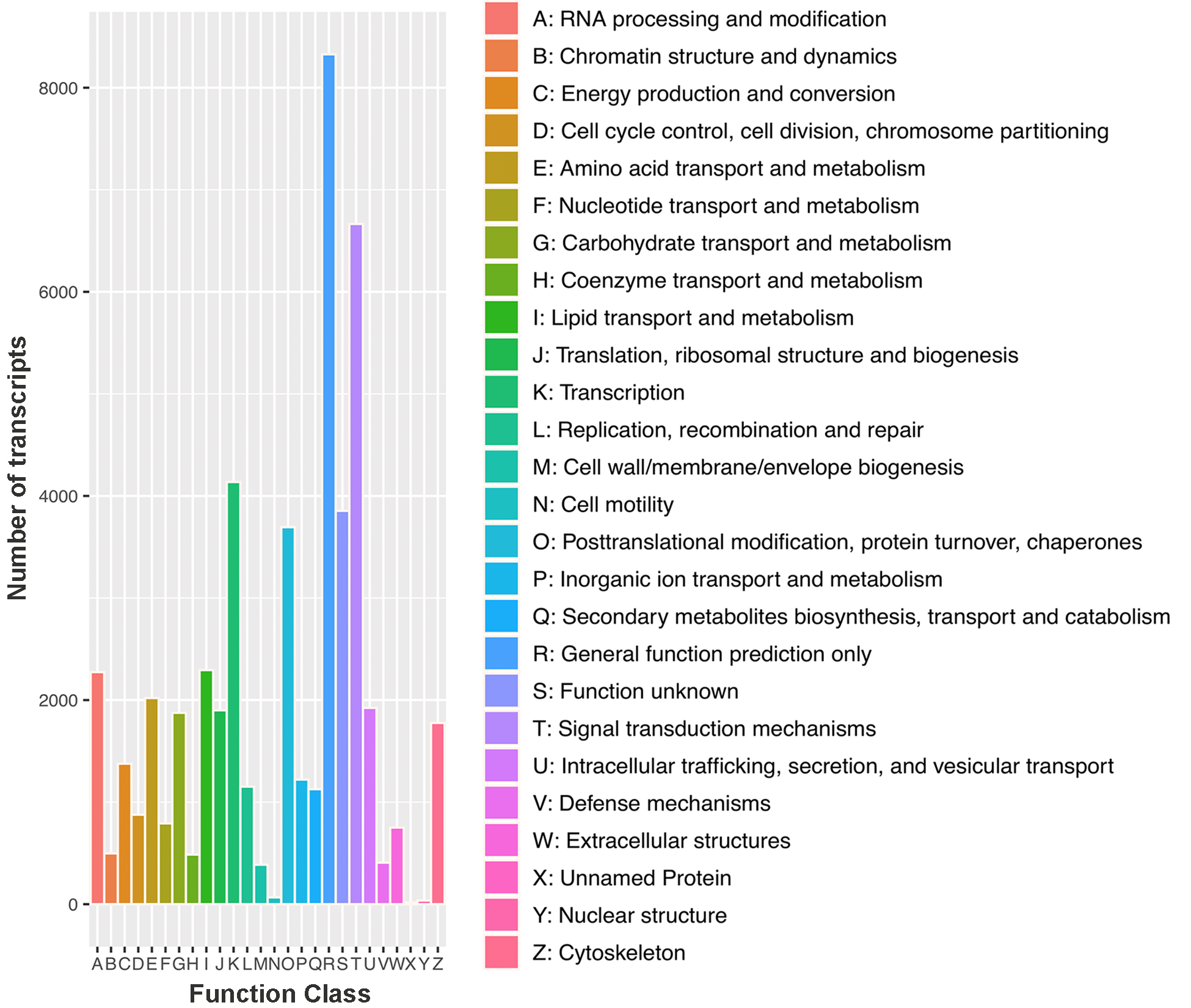

Supplement: S3 Fig — Among the 26 KOG function groups, the transcripts ‘General function prediction only’ was most represented (~16.68%) followed by ‘Signal transduction mechanisms’ (~13.35%). Percentages were calculated for each function class by dividing the total number of transcripts annotated to each class by the total number of transcripts annotated to KOG. (TIF) [file pone.0234903.s003.tif]

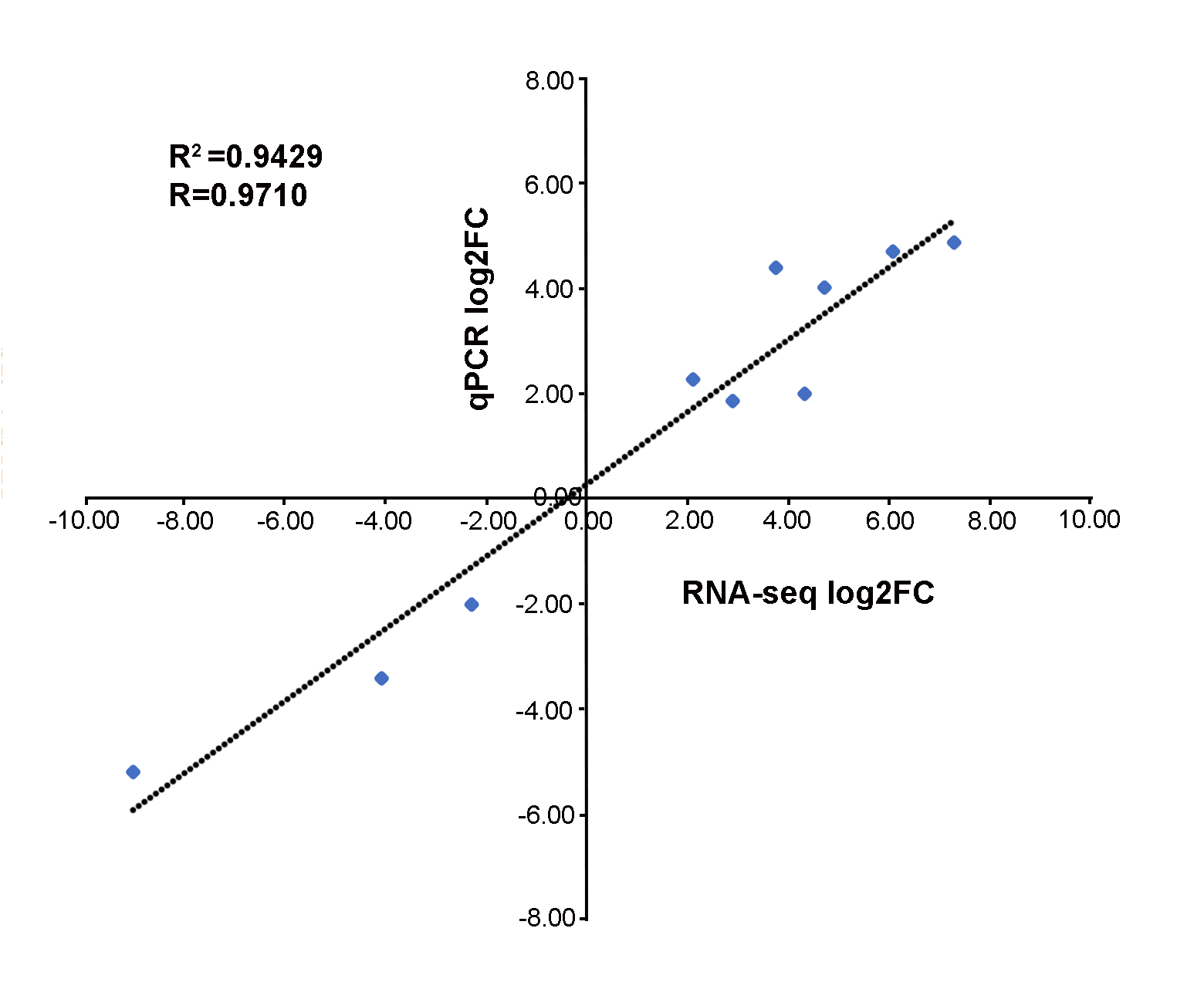

Supplement: S4 Fig — Scatter plot showing transcript expression in terms of log2-fold changes obtained from RNA-seq and RT-qPCR data for arbitrarily selected DETs. Each diamond represents a DET, N = 10. Linear regression analysis revealed high correlation between RNA-seq and RT-qPCR data (R2 = 0.9429, R = 0.9710). (TIF) [file pone.0234903.s004.tif]

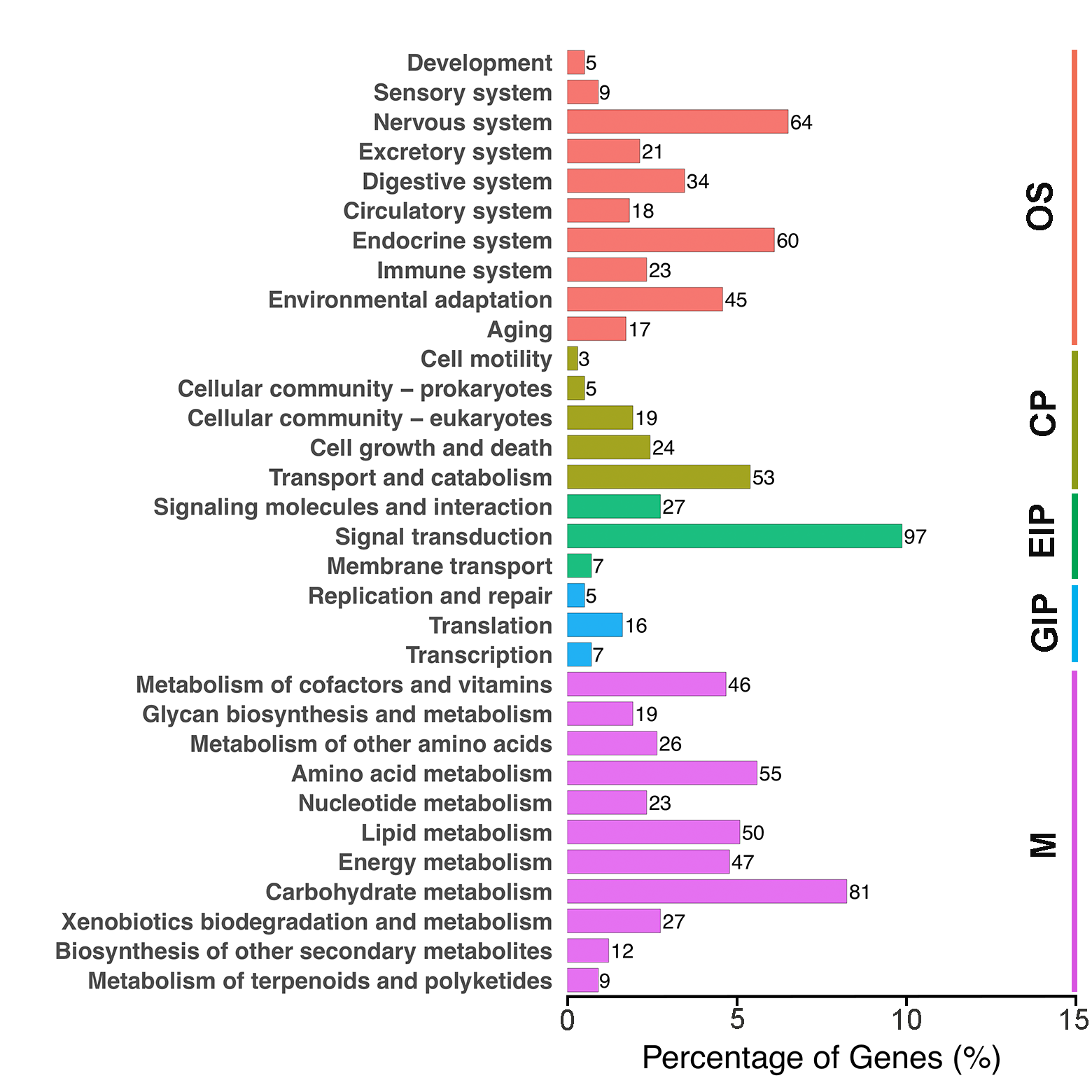

Supplement: S5 Fig — OS = Organismal systems, CP = Cellular Processes, EIP = Environmental Information Processing, GIP = Genetic Information Processing, M = Metabolism. Maximum number of DETs were identified to be associated with metabolic pathways, within which carbohydrate metabolism (~8.5%) and amino acid metabolism (~5.8%) were the top two pathways. (TIF) [file pone.0234903.s005.tif]

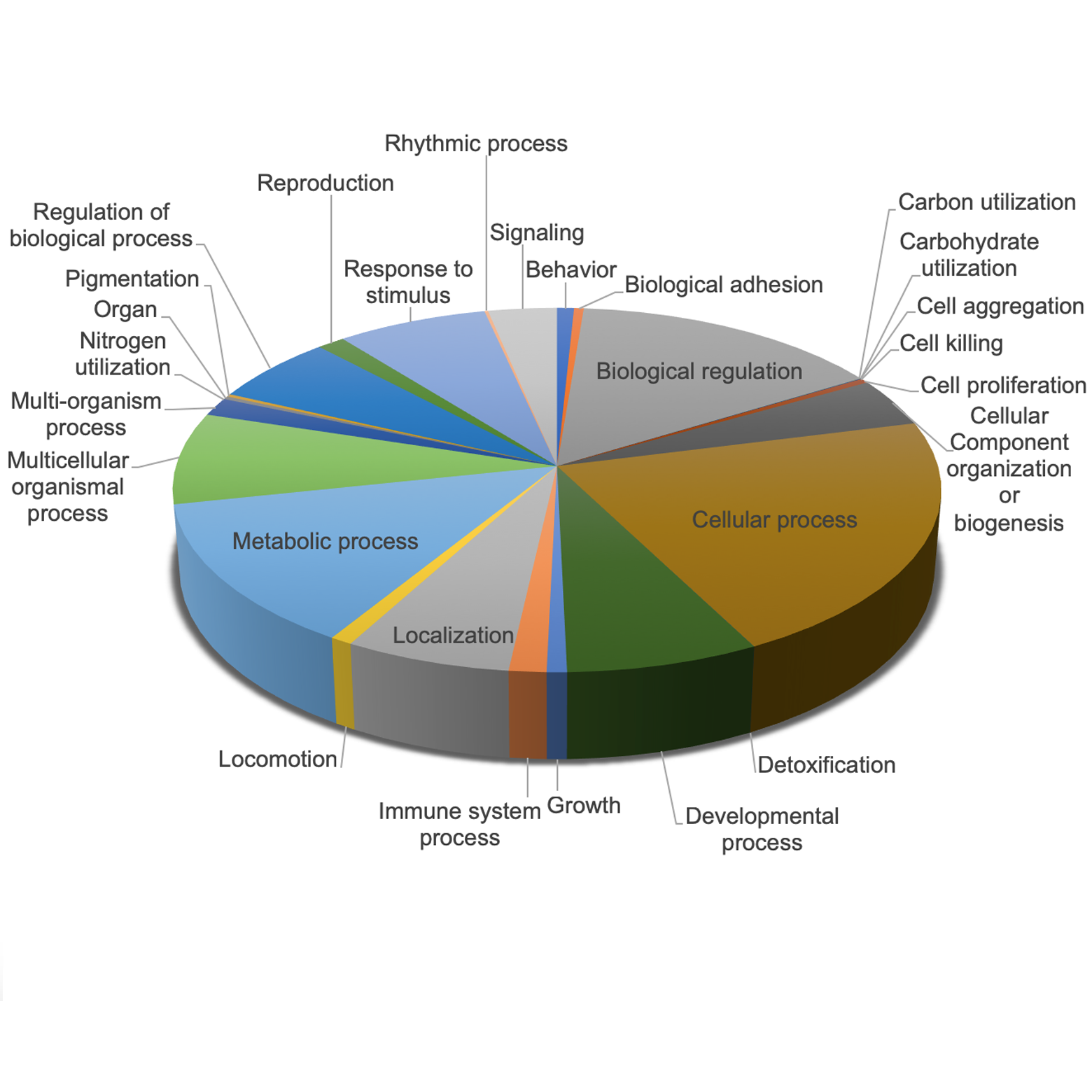

Supplement: S6 Fig — Pie chart represents the classification of transcripts in the functional category—biological process. (TIF) [file pone.0234903.s006.tif]
