# Supplementary material for: Transcriptome analysis of a social caterpillar, Drepana arcuata: De novo assembly, functional annotation and developmental analysis
Source: PLoS One. 2020 Jun 22;15(6):e0234903. doi: 10.1371/journal.pone.0234903 (PMC7307738; doi:10.1371/journal.pone.0234903)
Supplement: S1 Table — (DOCX) [file pone.0234903.s007.docx]

**S1 Table. Availability of data used in the transcriptomic analysis of larval *D. arcuata***

| **Data set** | **Accession number/File names** | |
| --- | --- | --- |
| Raw RNA-Seq reads | Bioproject PRJNA556910 | |
|  | **SRA Accession**  SRR9883279  SRR9883281  SRR9883284  SRR9883280  SRR9883282  SRR9883283 | **Biosample**  SAMN12402633  SAMN12402635  SAMN12402638  SAMN12402634  SAMN12402636  SAMN12402637 |
| Transcriptome assembly sequences | DDBJ/EMBL/GenBank under the accession GIKL00000000 | |
| Expression data for all transcripts | Gene Expression Omnibus (GEO) under the accession GSE146351 | |
