# Supplementary material for: Transcriptome analysis of a social caterpillar, Drepana arcuata: De novo assembly, functional annotation and developmental analysis
Source: PLoS One. 2020 Jun 22;15(6):e0234903. doi: 10.1371/journal.pone.0234903 (PMC7307738; doi:10.1371/journal.pone.0234903)
Supplement: S2 Table — (DOCX) [file pone.0234903.s008.docx]

**S2 Table. Transcript IDs and sequences of primers used for RT-qPCR validation of selected DETs in late vs early instars**

| **Transcript ID** | **Primer Sequence** | **NR ID** |
| --- | --- | --- |
| TRINITY_DN12902_c0_g1_i1 | F GGCACAAGAAATAGCAAAGC  R CAAGGCTTTCAATTCAGCAT | XP_022815561.1 |
| TRINITY_DN19051_c1_g1_i1 | F TAAAAAGGCGCAAATACAGG  R TTGGAGAAATGTTTCGTGGT | KPJ03807.1 |
| TRINITY_DN22716_c4_g2_i1 | F AATAAAAATCCGCTCCATCC  R GCTATAAACGTGGCGGATTA | XP_022816803.1 |
| TRINITY_DN18436_c1_g4_i1 | F CATGTCAGCGAAAACTTGTG  R AAAGCACGCTCAATCAGTTC | XP_021187481.1 |
| TRINITY_DN13131_c0_g1_i1 | F AACGTCGTTGTGAAAAGCTC  R GGTAATCCGTTGATTTGTCG | XP_012545354.1 |
| TRINITY_DN16368_c1_g3_i2 | F TCAGTGTCCATGCATTCGT  R ACATTCTGCGACCTTGGTT | XP_022826184.1 |
| TRINITY_DN17711_c2_g1_i9 | F AGCCCAGTTTCATGACCAA  R ACAAGTTGGTCGAGACGTT | XP_011562341.1 |
| TRINITY_DN13071_c0_g1_i1 | F AGAACACCGATCTTGCACA  R TCTTGTACGGTTGTTGGCA | PCG79677.1 |
| TRINITY_DN17510_c2_g2_i2 | F TGTATTGATGCGCCAGTCT  R ATGCCAACGTCATTGTCCT | KOB74604.1 |
| TRINITY_DN13765_c0_g1_i2 | F CGTAAAGTCCTCCCAGAGCG  R GCTACAGAATAAAACCATGTCCCG | NP_001127729.1 |
