# Supplementary material for: Transcriptome analysis of a social caterpillar, Drepana arcuata: De novo assembly, functional annotation and developmental analysis
Source: PLoS One. 2020 Jun 22;15(6):e0234903. doi: 10.1371/journal.pone.0234903 (PMC7307738; doi:10.1371/journal.pone.0234903)
Supplement: S3 Table — (DOCX) [file pone.0234903.s009.docx]

**S3 Table. Summary of RNA sequencing data and transcriptome assembly for early and late instar larvae**

| **Sequencing data** | | |
| --- | --- | --- |
|  | **Early instar** | **Late instar** |
| Total raw reads | 134,237,850 | 125,163,731 |
| Total clean reads | 108,028,166 | 102,984,128 |
| % GC | 48 | |
| **Transcriptome assembly statistics- Early instar** | | |
|  | **Transcripts** | **Unigenes** |
| Total number | 163,983 | 77,607 |
| N50 length | 2151 | 1556 |
| Median length | 559 | 356 |
| Average length | 1131.54 | 766.01 |
| Total assembled bases | 185,552,862 | 59,447,459 |
| % GC | 42.32 | |
| **Transcriptome assembly statistics- Late instar** | | |
|  | **Transcripts** | **Unigenes** |
| Total number | 166,348 | 83,125 |
| N50 length | 2149 | 1489 |
| Median length | 526 | 340 |
| Average length | 1105.73 | 729.13 |
| Total assembled bases | 183,936,505 | 60,608,956 |
| % GC | 42.89 | |
